# The effect of gastrointestinal microbiome supplementation on immune checkpoint inhibitor immunotherapy: a systematic review

**DOI:** 10.1007/s00432-023-04656-8

**Published:** 2023-03-16

**Authors:** Anjali Bhatt, Alyson Haslam, Vinay Prasad

**Affiliations:** 1 School of Medicine, Texas A&M Health Science Center, Bryan, TX 77807 USA; 2 Department of Epidemiology and Biostatistics, University of California San Francisco, 550 16th St, 2nd Fl, San Francisco, CA 94158 USA

**Keywords:** Gastrointestinal microbiome, Immune checkpoint inhibitor, Fecal microbiome transplants, Nivolumab, Ipilimumab, Pembrolizumab, GI microbiome, Systematic review

## Abstract

**Purpose:**

Gastrointestinal (GI) microbiome modulators, such as fecal microbiome transplants (FMTs), are being considered as supplements to standard immune checkpoint inhibitor (ICI) treatment to improve efficacy. This systematic review aims to assess the study design and outcomes of clinical trials that use FMTs to enhance ICI treatment.

**Methods:**

Systematic literature searches were conducted on PubMed and Embase using search terms that included names of ICIs and gastrointestinal microbiome. A first search identified interventional trials, and the second search identified interventional, retrospective, and observational studies.

**Results:**

The search for interventional trials produced 205 articles, 3 of which met the inclusion criteria. All studies had sample sizes ranging between 10 and 30 participants. 2 of the studies were single-arm studies with no control arm. One study reported an overall response rate (ORR) of 3 out of 15 (20%), a median progression-free survival (PFS) of 3 months, and a median overall survival (OS) of 7 months. The second study reported 1 complete response out of 10 (10%) and 2 partial responses out of 10 (20%). The third study reported an ORR of 58% vs. 20%, a median PFS of 12.7 months vs. 2.5 months in patients receiving nivolumab–ipilimumab plus CBM588 compared with patients receiving nivolumab–ipilimumab alone respectively, and an undefined median OS.

**Conclusion:**

Current studies on the microbiome modulators with ICI use are limited in study design. Future clinical trials should be randomized, use larger sample sizes, and use an appropriate control arm to better ascertain the clinical effect of the GI microbiome on ICI treatment.

**Supplementary Information:**

The online version contains supplementary material available at 10.1007/s00432-023-04656-8.

## Introduction

Immune checkpoint inhibitors (ICIs) are commonly used in the treatment of cancers, such as metastatic melanoma, renal cell carcinoma, and non-small cell lung cancer (Haslam and Prasad 2019); (Haslam et al. 2020). ICIs, which target programmed cell death-1 (PD-1), programmed cell death ligand-1 (PD-L1), and cytotoxic T-lymphocyte-associated protein-4 (CTLA-4), have extended survival in many tumor types, including metastatic melanoma that has historically had poor survival due to low response to traditional cytotoxic regimens. These mechanisms lead to the inability of T-lymphocytes to respond to and eliminate cancer cells from the body. Therefore, the mechanism of action of immune checkpoint inhibitors prevents T-lymphocyte inactivation and death.

However, many patients exhibit primary or acquired resistance to ICIs due to factors including low mutational burden, absence of tumor antigens, impaired cell signaling pathways, and the gastrointestinal (GI) microbiome (Bagchi et al. 2021). The GI microbiome consists of various microorganisms, including bacteria, fungi, and viruses, that play a role in physiologic functions, such as inflammation, immunity, and metabolism (Li et al. 2019); (Rezasoltani et al. 2021). Recently, GI microbiome modulators, such as fecal microbiome transplants (FMTs) and probiotics, have been considered as potential supplements to standard ICI treatment to improve efficacy and decrease drug resistance (Yi et al. 2018). This systematic review aims to assess the study design and outcomes of clinical trials that use FMTs and other GI microbiome modulators to enhance ICI treatment.

## Methods

### Search strategy

Two separate searches were conducted on PubMed and Embase databases. The first was to categorize the study designs of the first 100 results generated with search terms including immune checkpoint inhibitors and gastrointestinal microbiome. The second was to search for clinical trials that assess GI microbiome modulators as supplements to ICI therapy compared to ICI therapy alone.

A systematic search was conducted on July 11, 2022 on PubMed and Embase databases. The search terms for PubMed were (“Immune Checkpoint Inhibitors”[Majr]) OR “pembrolizumab” [Supplementary Concept]) OR “Nivolumab”[Mesh]) OR “cemiplimab” [Supplementary Concept]) OR “atezolizumab” [Supplementary Concept])) OR “avelumab” [Supplementary Concept]) OR “durvalumab” [Supplementary Concept]) OR “Ipilimumab”[Mesh]) AND “Gastrointestinal Microbiome”“[Mesh]”. The search terms for Embase were ‘immune checkpoint inhibitors’ or ‘checkpoint inhibitor’ or pembrolizumab or keytruda or nivolumab or opdivo or cemiplimab or libtayo or atezolizumab or tecentriq or avelumab or bavencio or durvalumab or imfinzi or ipilimumab or yervoy) and (‘gastrointestinal microbiome’ or microbiome)).mp. [mp = title, abstract, heading word, drug trade name, original title, device manufacturer, drug manufacturer, device trade name, keyword heading word, floating subheading word, candidate term word].

Another systematic search was conducted on April 28, 2022 on PubMed and Embase databases. The search terms for PubMed were “(“Immune Checkpoint Inhibitors”[Majr]) OR "pembrolizumab” [Supplementary Concept]) OR “Nivolumab”[Mesh]) OR “cemiplimab” [Supplementary Concept]) OR “atezolizumab” [Supplementary Concept])) OR “avelumab” [Supplementary Concept]) OR “durvalumab” [Supplementary Concept]) OR “Ipilimumab”[Mesh]) AND “Gastrointestinal Microbiome"[Mesh]”. The search terms for Embase were “((‘immune checkpoint inhibitors’ or ‘checkpoint inhibitor’ or pembrolizumab or keytruda or nivolumab or opdivo or cemiplimab or libtayo or atezolizumab or tecentriq or avelumab or bavencio or durvalumab or imfinzi or ipilimumab or yervoy) and (‘gastrointestinal microbiome’ or microbiome)).mp. [mp = title, abstract, heading word, drug trade name, original title, device manufacturer, drug manufacturer, device trade name, keyword heading word, floating subheading word, candidate term word]. The searches were limited to clinical trial or randomized controlled trial or controlled clinical trial or multicenter study or phase 1 clinical trial or phase 2 clinical trial or phase 3 clinical trial or phase 4 clinical trial.

Since most trials were single-arm trials, we also searched for FDA registration trials for the same tumor type and line of therapy.

### Inclusion/exclusion criteria

We included all articles from the first systematic search so that we could categorize study designs.

To assess clinical trial design and outcomes in the second systematic search, we included original articles that were in English and were interventional studies using human subjects. We excluded articles that were observational, retrospective, reviews, case reports, and not in English. Additionally, articles that discussed trials on animal subjects, such as mice, were not included due to the variability in study methodology and clinical endpoints between human and animal subjects. Articles were excluded if they were published in abstract form or did not provide sufficient information about the trial. Our primary interest was to focus on the effect of reestablishing the GI microbiome in the efficacy of immune checkpoint inhibitors in the treatment of cancer. Therefore, we excluded trials that focused on interventions that disrupt the GI microbiome, such as antibiotics.

### Data extraction

For each article identified in the second search, we extracted data that fit into four main categories: 1) article information, 2) patient population, 3) study design and quality, and 4) study outcomes. The article information included author, year published, and NCT number. The patient population included the patient’s diagnosis, cancer type and setting, and demographics of the patient population, such as median age of study participants and percent of male and female participants. Study design and quality factors included the year enrollment began, the intervention and control arms, sample size, endpoints, phase of trial, randomization, blinding, and funding. All results were reviewed by two authors (AB and AH). PRISMA (Preferred Reporting Items for Systematic Reviews and Meta-Analyses) guidelines were observed.

### Quality of studies

The Jadad score was used to assess the quality of the included controlled trials with a minimum of zero points and a maximum of five points (Berger and Alperson 2009). This method focuses on three components of clinical trials, which include masking, randomization, and accountability of patients, such as specifying reasons for withdrawals and dropouts. Points were given for meeting the three mentioned components and for being described and appropriate. Points were deducted for using inappropriate methods of randomization and/or masking. Scores were totaled based on ability to meet these criteria, and higher scores suggest better study design and quality.

### Statistical analysis

We used US Food and Drug Administration (FDA) data that reported the overall response rate (ORR) for clinical trials that studied pembrolizumab alone and nivolumab alone, respectively, in patients with un-resectable or metastatic melanoma. These data provided us a baseline comparator for outcomes with treatment using the drug alone versus treatment with FMT. A chi-square test was performed to see if there was a significant difference between FDA ORR and FMT ORR. An observed ORR was calculated using both the individual ORR outcomes from the pembrolizumab plus FMT trial and the nivolumab plus FMT trial. The expected ORR was calculated using FDA data on trials using pembrolizumab alone and nivolumab alone. Although the observed and expected outcomes are not directly comparable, the indication for each is similar in studying patients with metastatic melanoma. The chi-square test analysis was conducted using Microsoft Excel, version 16.51. Publicly available data without any patient identifiers were used in our analysis. Therefore, oversight was not required by an Institutional Review Board.

## Results

### Landscape of studies evaluating microbiome modification in combination with ICIs

**Fig. 1:**
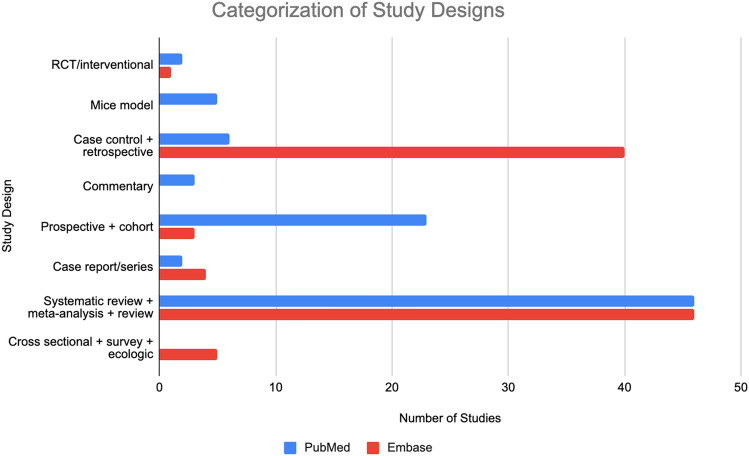
Study design categorization of the First 100 Results on Pubmed and Embase for studies evaluating gastrointestinal microbiome modulators with immune checkpoint inhibitors

We sought to ascertain the study design of microbiome studies indexed in 2 databases: PubMed and Embase. Up to 100 results were categorized by study design for a representative subset. As PubMed only generated 87 results, all 87 were included in the analysis. The most common study types for both PubMed and Embase were reviews (*n* = 42 vs 27, respectively). In PubMed, the next most common article types include prospective observational (*n* = 22), retrospective observational (*n* = 5), and mice models (*n* = 5). In Embase, the next most common study types include disproportionality analyses (*n* = 21), systematic reviews (*n* = 13), and retrospective observational (*n* = 10). Clinical trials were included as one of the least common study types in both PubMed and Embase (*n* = 2 vs 1, respectively). Thus, out of the 87 results in PubMed, 2.3% of results were clinical trials and 48.3% were reviews. Out of the 100 results included for Embase, 1.0% were clinical trials and 27.0% were reviews. A visual depiction of this information can be found in Fig. 1.

### Prospective experimental studies of microbiome manipulation in combination with ICIs

**Fig. 2:**
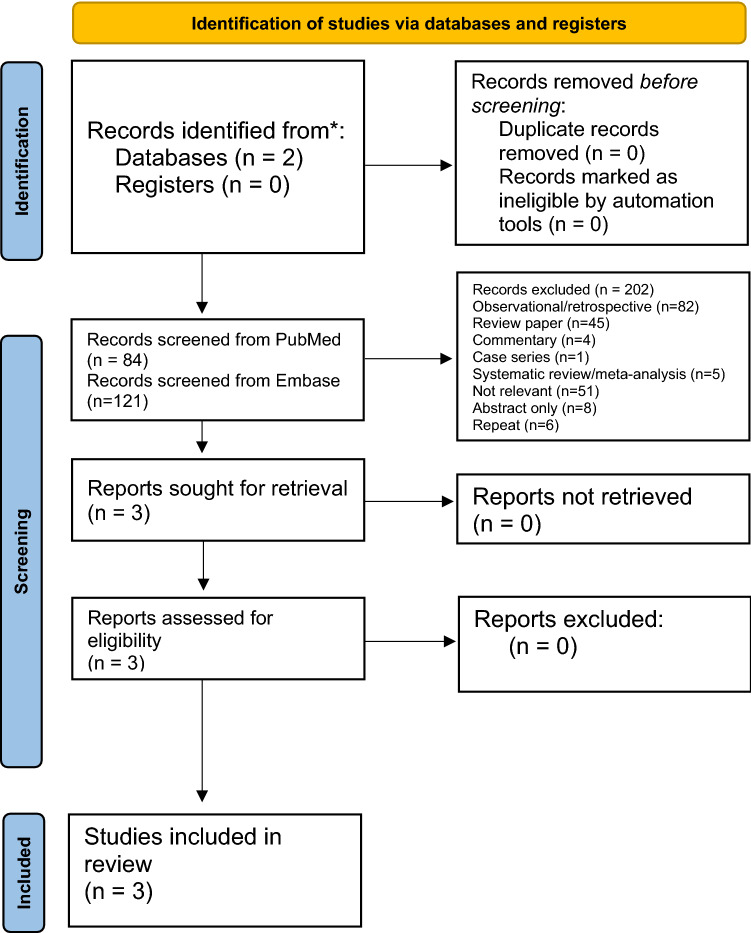
PRISMA diagram of systematic search results for studies evaluating gastrointestinal microbiome modulators with immune checkpoint inhibitors

Our secondary purpose was to summarize the evidence for microbiome manipulation on cancer outcomes in prospective studies. The search on PubMed and Embase that was limited to clinical trials generated 84 and 121 results, respectively, for a total of 205 results (Fig. 2). After screening these, based on title, abstract, and full text, a total of 202 articles were removed. Eighty-two were removed due to being an observational or retrospective paper, 45 were reviews, 4 were commentaries, 1 was a case series, 5 were systematic reviews or meta-analyses, 51 had no relevance to our desired question, 8 were in abstract form only, and 6 were duplicates.

For studies that reported demographic characteristics of study participants (Table 1), the median age of study participants was 66 years old (range = 66, *n* = 2 studies), the average percentage of male population was 71% (range = 70–72%, *n* = 2 studies), and the average percentage of female population was 29% (range = 28–30%, *n* = 2 studies). The remaining study in this analysis did not include demographic characteristics.

**Table 1:** Reported characteristics, demographics, and outcomes in studies evaluating gastrointestinal microbiome modulators with immune checkpoint inhibitors

	Davar (2021)	Baruch (2021)	Dizman (2022)
NCT #	NCT03341143	NCT03353402	NCT03829111
Phase of study	2	1	1
Study funder	Merck MSD	Ella Lemelbaum Institute for ImmunoOncology internal funds	Gateway for Cancer Research
Patient Population	Melanoma patients primary refractory to anti-PD-1 therapy	Metastatic melanoma with progression on at least one line of anti-PD-1 therapy; BRAF-V600E mutation; progression on BRAF-targeted therapy	Treatment-naive patients with metastatic renal cell carcinoma clear cell and/or sarcomatoid histology and intermediate or poor-risk disease
Cancer type & setting	PD-1 advanced melanoma	Metastatic refractory melanoma	Metastatic renal cell carcinoma
Years of data collection	2018–2020	2017–2021	2019–2022
Sample size (ss)	16	10	30, 29 in final analysis
Intervention arm & sample size (ss)	Single donor-derived FMT + pembrolizumab followed by additional pembrolizumab therapy every 3 weeks until disease progression or intolerable toxicity ss: 15	FMT (colonoscopy and oral stool capsules) followed by reinduction of anti-PD-1 therapy (nivolumab) ss: 10	CBM588, nivolumab, ipilimumab ss: 19
Control arm & sample size (ss)	N/A	N/A	nivolumab, ipilimumab ss: 10
Microbiome depletion phase?	No	Yes, orally ingested antibiotics (vancomycin and neomycin) for 72 h	No
Immune checkpoint inhibitor	Anti-PD-1	Anti-PD-1	Anti-PD-1 and anti-CTLA4
Primary endpoint(s)	ORR	FMT-related adverse events; proper implant engraftment	Change in Bifidobacterium composition of stool
Secondary endpoint(s)	Incidence of grade 3/4 toxicities, PFS, OS, change in T cell composition, change in innate/ adaptive immune system subsets, function of T cells	Changes in composition of immune cell population, changes in activity of immune cells	Change in Shannon index, best overall response, PFS
Median age of study participants	Unknown	66 years	66 years
% Male participants	Unknown	70%	72%
% Female participants	Unknown	30%	28%
ORR	3 out of 15 (20%)	1 CR out of 10 (10%) 2 PR out of 10 (20%)	11 patients (58%) receiving nivolumab–ipilimumab plus CBM588 compared with two patients (20%) receiving nivolumab–ipilimumab
Durable stable disease	3 out of 15 (20%)	N/A	N/A
Median PFS	All patients: 3 months Patients with stable disease: 14 months	Responders crossed PFS milestone of 6 months	12.7 months in patients receiving nivolumab–ipilimumab plus CBM588 versus 2.5 months in patients receiving nivolumab–ipilimumab
Median OS	All patients: 7 months Patients with stable disease: 14 months	N/A	Undefined (not reached) in intervention or control arm

Study characteristics, including funding, patient population, sample size, intervention and control arms, endpoints, outcomes, etc., are listed in Table 1. Two of the 3 trials studied the effects of FMT in addition to anti-PD-1 immunotherapy in patients with melanoma. One of these trials used pembrolizumab for patients with advanced melanoma (Davar et al. 2021), and another used nivolumab for patients with metastatic, refractory melanoma (Baruch et al. 2021). The third trial utilized an alternative microbiome modulator, CBM588, a probiotic that contains the Gram-positive bacterium, *Clostridium butyricum* (Dizman et al. 2022). This trial used CBM588 in addition to both anti-PD-1 and anti-CTLA4 immunotherapy, nivolumab, and ipilimumab, respectively, for patients diagnosed with metastatic renal cell carcinoma. The sample sizes for the 3 studies ranged from 10 to 30 participants. Two of the 3 studies (Davar et al. 2021); (Baruch et al. 2021) were designed as single-arm trials, with no control arm. The remaining third study (Dizman et al. 2022) had both an intervention and control arm, with a 2:1 randomization method. In one of the studies, a microbiome depletion phase was conducted before the intervention and control arm therapies were administered (Baruch et al. 2021). The microbiome depletion phase consisted of orally ingested antibiotics, including vancomycin and neomycin, for 72 h.

The most common clinical endpoints reported include ORR, median progression-free survival (PFS), and median overall survival (OS). The study utilizing FMT in addition to pembrolizumab for melanoma patients reported an ORR of 20% (Davar et al. 2021). The FDA reported an ORR of 24% with pembrolizumab alone (Keytruda label 2022). The study that included FMT in addition to nivolumab for melanoma patients reported 1 complete response (10%) and 2 partial responses (20%), for an ORR of 30%. The FDA registration trial for this tumor indication reported an ORR of 32%, with 4 complete responses (3%) and 34 partial responses (28%) for nivolumab alone (Opdivo label 2022). The third FMT study found that patients receiving nivolumab–ipilimumab plus CBM588 had a higher ORR than compared with patients receiving nivolumab–ipilimumab alone (58% versus 20%). The third study also reported a higher median PFS in patients receiving nivolumab–ipilimumab plus CBM588 compared with nivolumab–ipilimumab alone (12.7 months versus 2.5 months) (Dizman 2022). In a clinical trial comparing nivolumab plus ipilimumab versus sunitinib alone for advanced renal cell carcinoma (Checkpoint 214; NCT02231749), nivolumab plus ipilimumab had an undefined (not reached) median OS, an ORR of 42%, and median PFS of 11.6 months (Motzer et al. 2018). When comparing the results of the Checkpoint 214 trial with the third study conducted by Dizman et al., a discrepancy is seen between the ORR and PFS between the nivolumab–ipilimumab arm of each respective study (ORR: 42% vs 20%, respectively; median PFS: 11.6 months vs 2.5 months, respectively). This discrepancy may be due to differences in study design including sample size and randomization; however, it is a discrepancy that cannot be ignored as it calls into question the results of the intervention arm with CBM588. The remaining clinical endpoints of the included studies are reported in Table 1.

Overall, the included studies were limited in their quality and study design, with a median score of 1 point, mean of 1.67 points, and range of 1–3 points. More information regarding study design in terms of quality can be found in Table 2.

**Table 2:** Study design and quality; Jadad quality assessment scores for studies evaluating gastrointestinal microbiome modulators with immune checkpoint inhibitors

	Davar (2021)	Baruch (2021)	Dizman (2022)
NCT #	NCT03341143	NCT03353402	NCT03829111
Performed sample size calculation?	No	No	No
Randomization	None (only one arm)	None (only one arm)	Randomized 2:1
Masking	Open-label	Open-label	Open-label
Adequate power	No	No	Yes
Jadad quality assessment	1	1	3

Using a chi-square test testing reported response rates to established benchmarks from prior trials of ICIs alone, we found the following results: pembrolizumab: *χ*2 = 0.12, *p*-value =  0.73; nivolumab: *χ*2 = 0.12, *p*-value = 0.91, nivolumab-ipilimumab: *χ*2 = 2.03, *p*-value = 0.15

These results imply that there is no significant difference between the FMT ORR and FDA ORR, which suggests that GI microbiome supplementation may not lead to improved efficacy of ICI therapy based on current trial results and designs.

## Discussion

In this systematic review, we analyze the evidence landscape for the effect of gastrointestinal microbiome enhancement through FMT or probiotic supplementation on the effectiveness of ICI therapy.

We make two core findings. First, the landscape of studies shows excessive reliance on reviews, commentaries, case control, and mouse studies, and a paucity of prospective, experimental studies, and particularly randomized trials. The landscape suggests widespread enthusiasm, but to date, limited empirical verification. Second, among prospective experimental trials, evidence is equivocal. Response rates are modest, and randomization has occurred in only 1 instance, in a trial with limited power. A cross-trial comparison of RR in these non-randomized studies and established RRs in each tumor type for ICI alone should be taken solely as hypothesis-generating. These results cannot rule in or rule out a small and important difference in response rate, but do suggest that response rates appear to be in the same ballpark currently. As such, prospective, randomized evaluation is vital to separate potentially spurious inferences from causal effects.

It is also crucial to analyze these results in the context of the study design and quality of the individual clinical trials. Using the Jadad Quality Assessment scale, we found that 2 studies were limited in their study quality due to lack of randomization, masking, and therefore, they were given 1 point due to explanations on participants who withdrew or dropped out from the study (Davar et al. 2021); (Baruch et al. 2021). The remaining study was of moderate quality, as it lacked masking, but was randomized and explained reasons for participant withdrawal (Dizman et al. 2022). Additionally, 2 out of the 3 included studies had no control arm, and therefore, lacked a comparator (Davar et al. 2021); (Baruch et al. 2021). Thus, to compare these studies’ interventional arm with the drug alone, we accessed data on FDA-conducted clinical study results for use of pembrolizumab and nivolumab in the treatment of patients with metastatic melanoma. We acknowledge this comparison is hypothesis-generating only and can serve only to establish that response rates are in the same ballpark and further studies are needed.

For the studies that lacked a control arm, there was no difference between the ORR in the FDA registration trial and the ORR calculated with use of FMT plus pembrolizumab and nivolumab. A limitation of using data from FDA-conducted clinical trials is that there may be differences in study methodology and participants. For example, the FDA trials had different participant inclusion/exclusion criteria, larger sample sizes, were randomized, and different courses of treatment regimen. Nonetheless, it provides a ballpark sense for the baseline outcomes of potential control arms.

Many of the primary and secondary endpoints of these clinical trials focused on the safety, tolerability, adverse effects/toxicities, change in composition of the GI microbiome, and changes in immune cell composition. Clinical endpoints, such as ORR, PFS, and OS, were measured and were mainly included as secondary endpoints, but were emphasized in the discussion of outcomes. One reason for this may be that the topic is a relatively new avenue of research, which may increase the utility of information, such as adverse effects, mechanism of action, and effects on immune system.

Because of the varying trial design and endpoint analysis between the 3 included studies as indicated above, we stated the outcomes as indicated by each respective study rather than reporting them as pooled statistics. Pooled statistical analysis allows for a better evaluation of overall treatment effects and endpoints. However, we were limited by the number of studies in our analysis and by the low consistency in study design and measured endpoints.

Additionally, the relative novelty of this topic may also explain the lack of clinical trials with a study design that includes GI microbiome enhancement in addition to ICI treatment as an intervention in comparison to the number of review articles and observational studies. Many of the studies produced by our systematic search were prospective, retrospective, or included microbiome analysis as an exploratory endpoint, and therefore were excluded from our analysis of clinical trials.

Immuno-oncology (IO) is an emerging and revolutionary concept in cancer treatment. In fact, it has already revolutionized the cancer landscape with 43.6% of patients eligible for an IO drug. (Haslam and Prasad 2019); (Haslam et al. 2020). However, with its novelty, a multitude of unknowns regarding the application of IO in clinical practice arises. One of these unknowns is the development and utilization of appropriate predictive and prognostic biomarkers to guide clinical and therapeutic decisions in the realm of personalized oncology, with many patient-specific and tumor-specific variance (Rosellini et al. 2022). The predictive and prognostic value of novel biomarkers, including PD-L1 expression, tumor mutational burden (TMB), microsatellite instability (MSI), tumor microenvironment (TME), and gut microbiome, is fundamental in the therapeutic approach of utilizing ICI’s (Rosellini et al. 2022). Further study of these biomarkers has the potential to provide information regarding immune-related adverse effects (Massari et al. 2020) and early mortality. For example, positive and high levels of PD-L1 expression were shown to be associated with early mortality when treated with ICI alone, and this risk was reduced with a combination of ICI and other therapeutics (Viscardi et al. 2022). However, the use of these biomarkers in clinical practice remains unvalidated and should be further explored.

A strength of this study is that it is the first systematic review to study the effect of enhancing the gastrointestinal microbiome on the efficacy of ICI treatment. Additionally, we included only controlled clinical trials to best establish causality. Limitations of this study include the small number of included studies that fit our inclusion/exclusion criteria. This may be due to the relative novelty of the role of the microbiome in response to cancer therapeutics. Many studies that did study the role of the microbiome analyzed the effects of its depletion, for example via antibiotics. Our limited number of included studies may result in decreased reliability, external validity, and power, rendering it difficult to determine a true association between enhancing the GI microbiome and ICI treatment efficacy. Additionally, 2 out of the 3 studies in our systematic review lacked a control arm, were not randomized, and had small sample sizes, which may result in questionable internal validity of these studies. Due to these factors, readers should exercise caution when interpreting these results. Another limitation is that only 2 databases were used for our search, PubMed and Embase, and we were therefore limited to studies that were published in these databases. Additionally, some published abstracts met our inclusion/exclusion criteria in terms of study design and characteristics. However, these could not be included in our systematic review because the abstracts did not contain comprehensive information, including outcomes. Lastly, as 2 studies did not contain a control arm, FDA data were used both for comparison and statistical analysis. These studies are not directly comparable as they varied in sample size and patient population characteristics. However, we kept the indications and cancer types consistent during the comparisons and analysis.

## Conclusion

In conclusion, we found that studies evaluating GI microbiome supplementation to enhance ICI treatment efficacy in the treatment of cancers provide limited beneficial evidence. Further, these trials were limited in their ability to adequately answer this question. Randomized, masked, and controlled trials with larger sample sizes are needed to better imply causality between the microbiome and ICI treatment outcomes.

## Supplementary Information

Below is the link to the electronic supplementary material.

Supplementary file1 (DOCX 13 KB)

## Data Availability

Data for this analysis are included in the tables of the manuscript.
