# Supplementary material for: The effect of gastrointestinal microbiome supplementation on immune checkpoint inhibitor immunotherapy: a systematic review
Source: J Cancer Res Clin Oncol. 2023 Mar 16;149(10):7355–62. doi: 10.1007/s00432-023-04656-8 (PMC10374733; doi:10.1007/s00432-023-04656-8)
Supplement: Supplementary file 1 — Supplementary file1 (DOCX 13 KB) [file 432_2023_4656_MOESM1_ESM.docx]

Search strategy for studies evaluating and gastrointestinal microbiome on immune checkpoint inhibitor efficacy:

A systematic search was conducted on July 11, 2022 on PubMed and Embase databases. The search terms for PubMed were ("Immune Checkpoint Inhibitors"[Majr]) OR "pembrolizumab" [Supplementary Concept]) OR "Nivolumab"[Mesh]) OR "cemiplimab" [Supplementary Concept]) OR "atezolizumab" [Supplementary Concept])) OR "avelumab" [Supplementary Concept]) OR "durvalumab" [Supplementary Concept]) OR "Ipilimumab"[Mesh]) AND "Gastrointestinal Microbiome"[Mesh]". The search terms for Embase were 'immune checkpoint inhibitors' or 'checkpoint inhibitor' or pembrolizumab or keytruda or nivolumab or opdivo

or cemiplimab or libtayo or atezolizumab or tecentriq or avelumab or bavencio or durvalumab or imfinzi or ipilimumab or yervoy) and ('gastrointestinal microbiome' or microbiome)).mp. [mp=title, abstract, heading word, drug trade name, original title, device manufacturer, drug manufacturer, device trade name, keyword heading word, floating subheading word, candidate term word].

Another systematic search was conducted on April 28, 2022 on PubMed and Embase databases. The search terms for PubMed were “("Immune Checkpoint Inhibitors"[Majr]) OR "pembrolizumab" [Supplementary Concept]) OR "Nivolumab"[Mesh]) OR "cemiplimab" [Supplementary Concept]) OR "atezolizumab" [Supplementary Concept])) OR "avelumab" [Supplementary Concept]) OR "durvalumab" [Supplementary Concept]) OR "Ipilimumab"[Mesh]) AND "Gastrointestinal Microbiome"[Mesh]”. The search terms for Embase were “(('immune checkpoint inhibitors' or 'checkpoint inhibitor' or pembrolizumab or keytruda or nivolumab or opdivo or cemiplimab or libtayo or atezolizumab or tecentriq or avelumab or bavencio or durvalumab or imfinzi or ipilimumab or yervoy) and ('gastrointestinal microbiome' or microbiome)).mp. [mp=title, abstract, heading word, drug trade name, original title, device manufacturer, drug manufacturer, device trade name, keyword heading word, floating subheading word, candidate term word]. The searches were limited to clinical, randomized controlled, or controlled clinical trials of any phase.
